# Twenty-Eight Orders of Parametric Resonance in a Microelectromechanical Device for Multi-band Vibration Energy Harvesting

**DOI:** 10.1038/srep30167

**Published:** 2016-07-22

**Authors:** Yu Jia, Sijun Du, Ashwin A. Seshia

**Affiliations:** 1 Nanoscience Centre, University of Cambridge, Cambridge, CB3 0FF, United Kingdom; 2 Department of Mechanical Engineering, University of Chester, Chester, CH1 2NF, United Kingdom

## Abstract

This paper contends to be the first to report the experimental observation of up to 28 orders of parametric resonance, which has thus far only been envisioned in the theoretical realm. While theory has long predicted the onset of *n* orders of parametric resonance, previously reported experimental observations have been limited up to about the first 5 orders. This is due to the rapid narrowing nature of the frequency bandwidth of the higher instability intervals, making practical accessibility increasingly more difficult. Here, the authors have experimentally confirmed up to 28 orders of parametric resonance in a micromachined membrane resonator when electrically undamped. While the implication of this finding spans across the vibration dynamics and transducer application spectrum, the particular significance of this work is to broaden the accumulative operational frequency bandwidth of vibration energy harvesting for enabling self-powered microsystems. Up to 5 orders were recorded when driven at 1.0 g of acceleration across a matched load of 70 kΩ. With a natural frequency of 980 Hz, the fundamental mode direct resonance had a −3 dB bandwidth of 55 Hz, in contrast to the 314 Hz for the first order parametric resonance; furthermore, the half power bands of all 5 orders accumulated to 478 Hz.

Resonance is classically associated with the oscillatory amplitude build-up induced by a directly forced excitation of a system at a specified input frequency. Resonant amplification has wide implications for a multitude of applications in sensors, actuators as well as vibration energy harvesting; however, it is not limited only to the convention of direct excitation. For instance, other resonant phenomena, including parametric resonance^1^, have been researched with potential advantages in achieving higher mechanical amplification, thermo-mechanical noise squeezing^2^ and exceptional input-to-output sensitivity^3^. Parametric resonance arises from driving forces that induce a periodic variation in at least one of the system parameters, thus triggering an internal build up of energy, as opposed to the externally forced response of a mass-spring-damper system^4^,^5^.

One of the earliest recorded reports of this peculiar vibration phenomenon dates back to Michael Faraday in 1831^6^ upon observing a vertically oscillating cylinder on the surface of a fluid that had half the frequency of the excitation. Studies on the underlying mathematics were initiated by Mathieu^7^ and further established by the likes of Floquet^8^ and Hill^9^. Experimental investigations were carried out by Lord Rayleigh (J. Strutt) in 1880’s using vibrating strings and wave propagation apparatus^10^,^11^.

Traditionally, the study of parametric resonance has circled around its control and prevention in regards to structural failure^12^,^13^; as growing oscillation amplitude, en route to chaos, could accumulate to significantly larger amplitudes than direct resonance^5^. This also implies that it could act as a more effective mechanical amplifier for a given transducer, as previously demonstrated in the context of microelectromechanical (MEM) gyroscopes and mass sensors^14^,^15^. It has also been explored for vibration energy harvesting as a means to accumulate more mechanical energy^16^; and passive design approaches have been developed^17^ to overcome the critical initiation threshold excitation amplitude^1^, which would otherwise limit its onset.

Parametric resonance is distinct due to an instability phenomenon governed by the Mathieu function (equation 1)^4^, which is named after the elliptical membrane problem studied by M.E. Mathieu^7^.


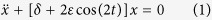


where, *δ* and *ε* are generic parameters relating to the square of natural frequency and the parametric excitation amplitude respectively. An instability chart, sometimes known as the Strutt diagram, can be plotted (see Fig. 1) from *δ* and *ε* to illustrate the regions where parametric resonance can be activated.

The diminishing size of each tongue tip towards the right-hand-side of the graph represents the fast narrowing trend in the frequency bandwidth of the higher order instability regions^18^,^19^. Therefore, the observation of higher orders has traditionally been elusive and only the first few orders have been experimentally confirmed for a classical macro-scale system^5^,^12^.

At the micro-scale, up to 4 orders for a nanowire mechanical system was observed^20^, while micro-fabricated MEM cantilever-based resonators unveiled up to 5 orders of parametric resonance^21^,^22^. The lower damping and relatively wider absolute frequency bandwidth in these microscopic systems facilitated the practical feasibility of the experimental measurements of the higher orders. In terms of topology, a circular plate design has been demonstrated to unveil higher orders at the macro-scale^23^.

This paper contends to be the first to report the experimental observation of at least 28 orders of confirmed parametric resonance. The opening up of these higher orders makes it possible to experimentally investigate and validate the current theory within structural dynamics research. Furthermore, a wider context can also be established in research areas such as microelectromechanical sensors and transducers to capture its engineering application. More specifically for vibration energy harvesting, this paper demonstrates a significant broadening of the accumulative frequency bandwidth, providing a practical pathway towards the potential realisation of vibration-powered smart microsystems.

## Design, Apparatus and Method

### Device

The design adopted was that of a circular disk with a centred circular mass (Fig. 2a). Design inspiration was drawn from macroscopic circular plates^23^, the membrane design used by Mathieu^7^ and membrane-based musical instruments such as the timpani. Such a microscopic thin membrane typically has notable amplitude dependent geometric nonlinearity^24^, which is further enhanced by the presence of the proof mass.

The MEMS membrane device has a combined radius of 3.5 mm, including a circular proof mass of radius 1.5 mm. The regions of the membrane, not housing the proof mass, is mainly comprised of a 0.5 μm AlN (aluminium nitride) piezoelectric thin film deposited by reactive sputtering onto a 10 μm doped silicon device layer, while the proof mass is constructed from an unetched suspended silicon substrate. The active piezoelectric regions were completed by a top metal layer comprised of 20 nm Cr and 1000 nm Al deposited by beam evaporation. Within this active region of the membrane, there are two rings of strain: the inner ring representing the bending strain and the outer ring of anchor strain as can be seen from Fig. 2a.

An outline of the piezoelectric MEMS fabrication process is shown in Fig. 2b. Wire bonds were connected to the inner and outer electrode rings in order to complete the circuit around the piezoelectric transducer. The fabricated MEMS chip shown in Fig. 2c is fixed to a leadless chip carrier (LCC), which is itself laser back etched in order to accommodate unconstrained travel of the resonator proof mass. The overview of the experimental apparatus is included in the supplementary material and is based on previous work^16^,^17^,^22^.

### Analytical

#### Duffing Mathieu Link

It has been previously shown that Duffing oscillators driven by an external periodic excitation can, under the right conditions, describe bifurcations in response leading to instability^25^,^26^. Specifically, in the presence of small uncertainties in the system parameters or noise^25^, the perturbation solution for the periodically driven Duffing oscillator can be seen to satisfy the Mathieu equation^26^. An externally driven Duffing oscillator can be modeled by equation 2.


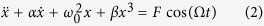


where, *α* is the mass normalised viscous damping, *ω*_0_ is natural frequency, *β* is the mass normalised Duffing coefficient, *F* is the mass normalised forcing amplitude, Ω is forcing frequency, *x* is displacement and *t* is time domain. A describing function approach can be adopted for small nonlinearity as shown in equation 3.


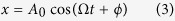


where, response amplitude *A*_0_ and phase angle *ϕ* can be found by substituting this solution back into equation 2, as displayed in equation 4.


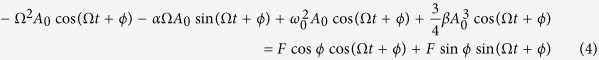


Equating terms in 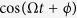 and 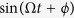 leads to equations 5 and 6.


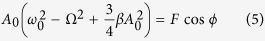



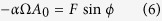


Therefore, *A*_0_ (equation 7) can be derived by squaring and adding equations 5 and 6; and *ϕ* (equation 8) can be derived by dividing equation 6 by equation 5.


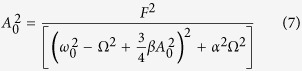



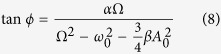


Now if we look for a solution for *x*(*t*) represented by a small perturbation *η*, we get *x*(*t*) = *x*_0_ + *η*, and by substituting this into equation 2, we get equation 10 (while dropping the high order terms).


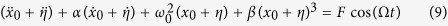



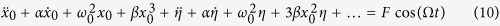


Assuming 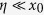, and using the fact that equation 3 is a solution of equation 2, equation 13 can be derived.


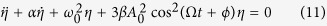



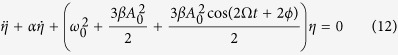



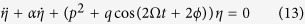



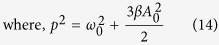



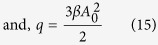


Equation 13 is a damped form of the Mathieu equation, previously shown in equation 1. Therefore, this establishes the link between a periodically forced Duffing equation and the Mathieu equation in the presence of noise. Physically, the periodic modulation in spring stiffness represents the parametric excitation, which would yield parametric resonance under the right frequency and amplitude conditions.

#### Disk membrane

The disk membrane can thus be reduced to a second order mass-spring-damper model governed by either equation 16 (when directly excited) or equation 17 (a variation of the damped Mathieu equation for parametric excitations). Non-linear terms are incorporated into equation 17 to empirically model amplitude saturation following excitation conditions that lead to unstable amplitude growth defined by equation 13.


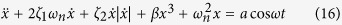



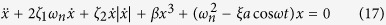


where, *ζ*_1_ is the viscous damping ratio, *ζ*_2_ is the nonlinear quadratic damping coefficient, *ξ* is a standard coefficient relating the external excitation to the parametric excitation, *a* is the acceleration amplitude, *ω* is the excitation frequency, *ω*_*n*_ is the natural frequency and *t* is the time domain. All parameters are mass normalised.

The adjusted natural frequency of a circular membrane with significant plate mass and a circular centred mass is shown in equation 18. The derivation is based on^27^ and is included in the supplementary document.


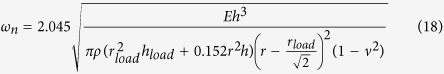


where, *E* is the elastic modulus, *h* is the thickness, *ρ* is mass density, *r* is the total radius, *r*_*load*_ is the radius of the proof mass and *ν* is the Poisson’s ratio.

Parametric resonance onsets when the system operates within the Mathieu instability regions, which corresponds to the frequency vicinities of 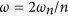; where *n* is a positive integer representing the order number. Therefore, *n* = 1 represents the first order or principal parametric resonance where *ω* is twice that of *ω*_*n*_. Generally, higher the order number, narrower the instability region; while increasing excitation amplitude or lowering damping would open up deeper access into the instability zones. However, regardless of the order number *n*, response frequency is always precisely *n*/2 times the excitation frequency *ω*.

### Simulation

The response of the system can be ascertained through numerical simulation and results are provided for representative choices of parameters in the supplemental document. In summary, it can be noted that the direct resonant build up is more immediate when compared to that of parametric. Another distinct characteristic of parametric resonance is the slope of the rate of change of the oscillatory amplitude build up. Furthermore, the response frequency of all orders of parametric resonances are always at *ω*_*n*_, in agreement with the theory. For instance, the excitation frequency of the first order is twice that of its response frequency.

## Experimental Results

### Higher order response

Under minimum damping conditions (electrically undamped), up to 28 orders of confirmed parametric resonance were experimentally observed for the MEMS disk resonator. Figure 3 illustrates the excitation frequency ranges where all 28 orders were measured, which is in close agreement to the theory. Further measurement details are included in supplemental material.

It can be noted that with higher orders, the bandwidth rapidly diminishes and becomes practically more difficult to trigger. Beyond the 28th order, the bandwidth becomes too narrow to scan due to the practical frequency of the shaker. From about the 8th order onwards, the instability intervals also become very small and multiple potentially operational frequency bands become closely populated. The onset of parametric resonance is dependant on the initial condition and the direction of the frequency sweep.

**Figure 1:**
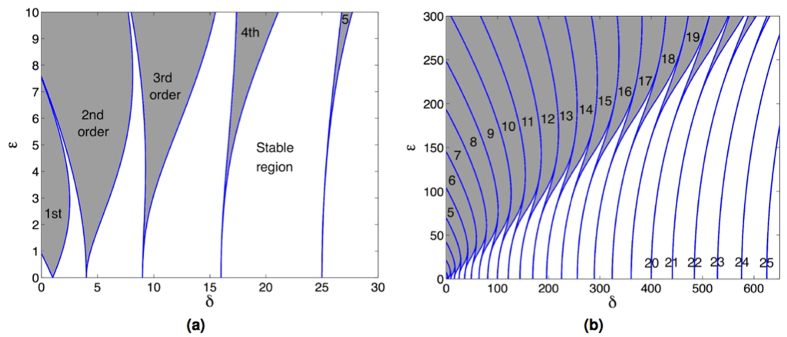
Strutt diagram showing the stable and unstable regions of the Mathieu equation. Shaded areas represent the instability regions enveloped within the blue curves, which are the loci of the parameters *δ* and *ε*. (**a**) 5 orders and (**b**) 25 orders of parametric resonance.

**Figure 2:**
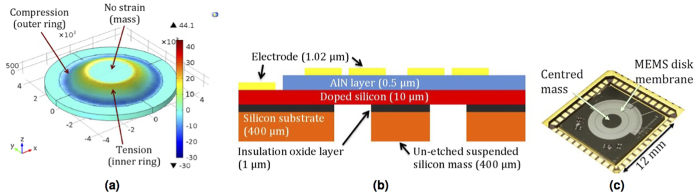
MEMS piezoelectric disk membrane resonator. (**a**) 3D CAD design rendition, (**b**) stack of materials for the MEMS process and (**c**) top side of wire bonded MEMS chip.

**Figure 3:**
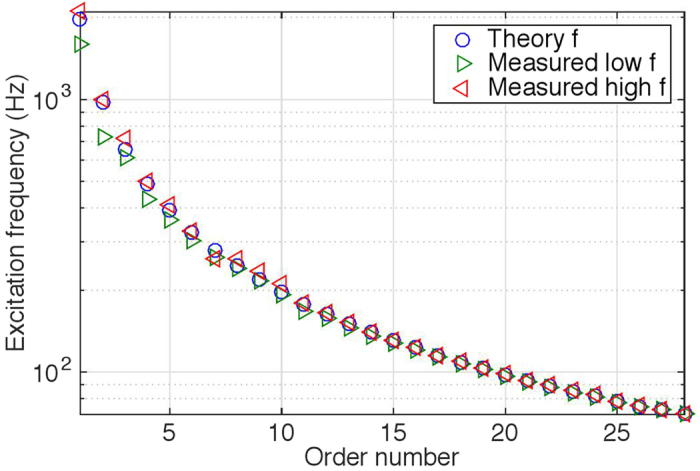
Measured excitation frequencies of 28 orders of parametric resonance compared to theoretical prediction. The measured low *f* and high *f* refer to the lower and higher instability boundaries of each order.

**Figure 4:**
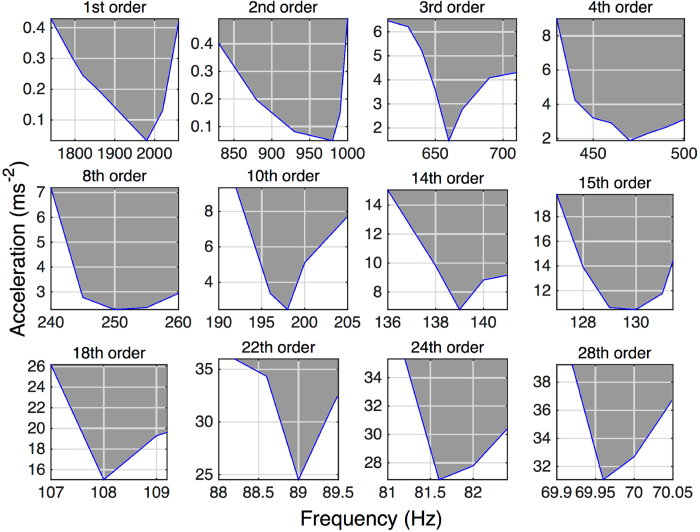
A representation of the experimentally observed instability regions for selected orders. The shaded regions are where the corresponding order of parametric resonance can be activated/maintained.

Figure 4 presents an experimentally recorded variant of the Strutt diagram for selected orders of parametric resonance. The charts were plotted for the boundary regions where the parametric resonant response at a particular frequency starts to onset or begins to decay. The downwards pointed triangular shapes are in general agreement with the tongue-shapes from the Strutt diagram (Fig. 1b). It can be seen that the higher order instabilities are centred around integer sub-multiples of twice the natural frequency with the threshold excitation required to trigger instability progressively increasing while the range of excitation frequencies about which the instability is observed progressively narrows.

**Figure 5:**
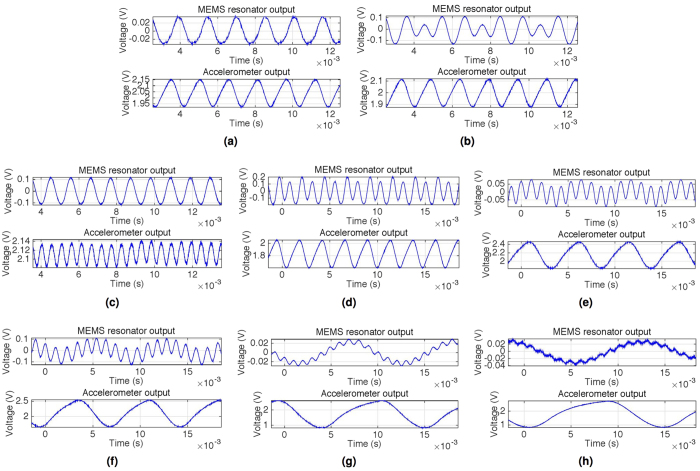
Selected orders of measured parametric resonance, showing both drive acceleration and MEMS resonator response. Upon attaining the initiation threshold amplitude, the specific order of parametric resonance onsets. (**a**) In frequency vicinity of 3rd order parametric resonance, driven at 4.0 ms^−2^ (below critical amplitude threshold): non-resonant direct response, (**b**) In frequency vicinity of 3rd order parametric resonance, driven at 4.2 ms^−2^ (above critical amplitude threshold): onset of parametric resonant response, (**c**) 1st order: drive frequency is twice the response, (**d**) 4th order: response frequency is twice the drive, (**e**) 10th order: response frequency is 5 times the drive, (**f**) 14th order: response frequency is 7 times the drive, (**g**) 22nd order: response frequency is 11 times the drive, (**h**) 28th order: response frequency is 14 times the drive.

Some of the criteria for identifying parametric resonance include the sudden onset and exit of the resonant regime around the boundary of instability regions, and the presence of a damping dependent initiation threshold amplitude below which parametric resonance cannot be achieved. These criteria were all experimentally consistent throughout the higher orders. Figure 5 provides further evidence that the observed time-domain response for a given excitation frequency is consistent with the onset of parametric resonance. Figure 5a shows the non-resonant direct response when the system is driven at 4.0 ms^−2^ within the vicinity where the 3rd order is theoretically expected. As the acceleration was increased to 4.2 ms^−2^, Fig. 5b shows a drastically higher response amplitude with the onset of 3rd order parametric resonance. While the non-resonant direct output has a response frequency identical to the excitation, the third order exhibits 3 oscillations of response per 2 oscillations of input. Also, each successive oscillation is no longer entirely symmetrical to the previous oscillation, as the system tries to ‘push in’ a third oscillation between the usual two oscillations.

The time-domain response of several other selected orders are also shown in Fig. 5. Regardless of the excitation frequency *ω*, as long as parametric resonance onsets, the response frequency is always at 0.5*n* multiples of *ω* (i.e., around *ω*_*n*_). While the parametric resonance dominates at the first few orders, the higher orders (starting from around the 8th) exhibit a non-resonant direct output as the baseline response while the higher frequency parametric response sits on top of this oscillating baseline as shown in Fig. 5e,f. Furthermore, this non-resonant output begins to dominate over parametric resonance at even higher orders, as illustrated by the manifestation of small parametric ripples sitting on top of the baseline in Fig. 5g,h. This is partly due to lower energy of the higher orders as well as the larger directly forced amplitude at lower frequencies. However, the frequency ratio signature of these higher order (ripples) still remain clear.

### Multi-band vibration energy harvesting

**Figure 6:**
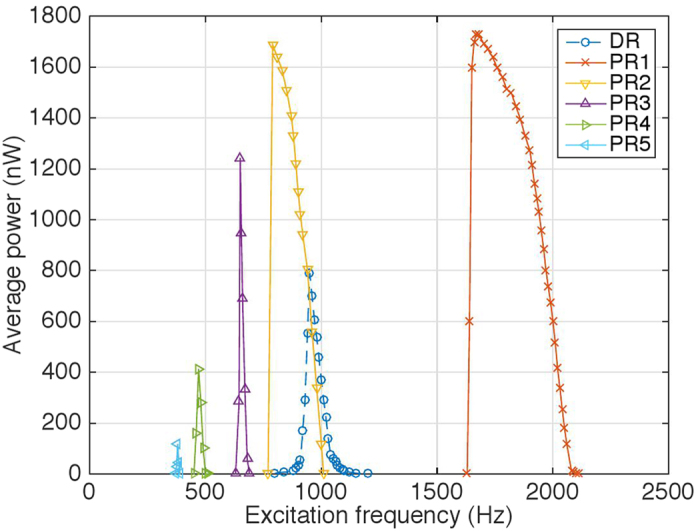
Frequency domain power output at 1.0 g of acceleration. DR (direct resonance) and PR (parametric resonance) and numeral represents order number.

Figure 6 presents the frequency domain power response (across a matched electrical load of 70 kΩ) of the piezoelectric membrane when subjected to 1.0 g of acceleration. Up to 5 orders of parametric resonance were recorded; along with the directly excited fundamental resonant mode, which demonstrated spring softening Duffing behaviour. Therefore, the onset of all 6 resonances recorded were sensitive to initial conditions.

An average power output of 0.79 μW and half power bandwidth of 55 Hz was recorded for the 1st mode direct resonance, 1.73 μW and 314 Hz for the 1st order parametric resonance as well as 1.69 μW and 135 Hz for the 2nd order (power output details for higher orders are provided in supplementary). Although observable, peaks higher than the 3rd order were both smaller and narrower than the 1st mode direct resonance. Nonetheless, the presence of the higher orders help open up multi-frequency bands, which would otherwise be non-existent.

Therefore, this contributes to the significantly larger accumulative half power bandwidth (478 Hz) of the parametric resonator for the purpose of vibration energy harvesting. Furthermore, if the half power point of the direct resonant peak was taken as the bandwidth reference, the accumulated operational frequency bandwidth of the parametric resonant regimes sums up to over 10 times wider (582 Hz) than the direct peak.

The presence of multiple orders of parametric resonances provide both broader absolute bandwidth and multiple operational zones, which help to improve the likelihood of ambient vibration activating one of the resonant regimes. Overall, coupled with the conventional direct resonant regime, this provides a pathway towards capturing a much wider spectrum of the available vibration energy.

## Conclusion and Future Work

This paper, employing a MEMS circular disk membrane resonator, reports the experimental observation of up to 28 orders of parametric resonance and thus confirming the existence of these theoretically predicted higher order Mathieu instability zones. For the application of vibration energy harvesting (electrically damped to extract energy), up to 5 orders were recorded at 1 g of acceleration; with peak power of 3.46 μW and −3 dB band of 314 Hz for the 1st order as well as an accumulative −3 dB band of 478 Hz, in contrast to the 1.58 μW and 55 Hz for the conventional direct resonant peak.

Future and ongoing work attempts to optimise the fabrication process and vacuum packaging. Unlike direct resonance, both the response amplitude and the frequency bandwidth for parametric resonance increase with decreasing damping^22^. Therefore, this approach helps circumvent the dilemma of compromising between resonant amplification and frequency bandwidth for classical resonators. Also, the precise response frequency to excitation frequency ratios, coupled with the sensitive boundary conditions associated with the higher orders have interesting implications for other sensing applications. Furthermore, the results presented here lays the foundation to experimentally investigate and validate the current theory for higher order instabilities and enable physical insight into the higher order nonlinear effects operative in such systems.

## Additional Information

**How to cite this article**: Jia, Y. *et al*. Twenty-Eight Orders of Parametric Resonance in a Microelectromechanical Device for Multi-band Vibration Energy Harvesting. *Sci. Rep.* **6**, 30167; doi: 10.1038/srep30167 (2016).

## Supplementary Material

Supplementary Information
