# Supplementary material for: Twenty-Eight Orders of Parametric Resonance in a Microelectromechanical Device for Multi-band Vibration Energy Harvesting
Source: Sci Rep. 2016 Jul 22;6:30167. doi: 10.1038/srep30167 (PMC4957125; doi:10.1038/srep30167)
Supplement: Supplementary Information [file srep30167-s1.pdf]

Supplementary Document for

Twenty-Eight Orders of Parametric Resonance in a Microelectromechanical Device for Multi-band Vibration Energy Harvesting

Yu Jia[1, 2, *], Sijun Du[1, -], and Ashwin A Seshia[1, +]

[1]Nanoscience Centre, University of Cambridge, Cambridge, CB3 0FF, United Kingdom
[2]Department of Mechanical Engineering, University of Chester, Chester, CH1 2NF, United Kingdom
[*]yu.jia.gb@ieee.org
[-]sd672@cam.ac.uk
[+]aas41@cam.ac.uk

ABSTRACT

This is the supplementary document to the paper titled, 'Twenty-Eight Orders of Parametric Resonance in a Microelectromechanical Device for Multi-band Vibration Energy Harvesting'.

Design, Apparatus and Method

Device

The design employed in this study is that of a microfabricated thin membrane (figure 1), which can yield geometric nonlinearity.[1] On the onset of this nonlinearity can result in Mathieu instability[2] through the periodic modulation of the spring restoring force. Additionally, MEMS devices[3] have been previously demonstrated to provide more accessibility to higher orders due to its relatively wider instability intervals and lower damping.

As illustrated from figures 1c and 1d, during each half cycle of the oscillation, the inner ring and outer ring of the disk membrane resonator experience opposing mechanical strain (compression and tension). Therefore, two separate rings of electrodes are attached as per the strain distribution according to the COMSOL solid mechanics simulation. An inner ring electrode creating a piezoelectric area for harvesting bending strain and an equivalent outer ring is responsible for the anchor strain.

Analytical

Disk membrane

The natural frequency of a plain circular disk membrane is given by equation 1.[4]

$$\omega_n = B\sqrt{\frac{Eh^2}{\rho d^4(1-\nu^2)}} \tag{1}$$

where, ω_n is the natural frequency, E is the elastic modulus, h is the thickness, ρ is the density, d is the diameter, ν is the Poisson's ratio and B is a coefficient determining the mode of resonance. The fundamental mode (01 mode) is when $B = 11.84$ as shown in equation 2 and the second resonant mode (11 mode) is listed in equation 3. The 01 mode, where the membrane oscillates up and down with no nodal diameter and 1 nodal circle, is the focus of this paper.

$$\omega_n = 11.84\sqrt{\frac{Eh^2}{\rho d^4(1-\nu^2)}} \quad ; \text{where, } n = 1 \quad \text{for 01 mode} \tag{2}$$

$$\omega_n = 24.61\sqrt{\frac{Eh^2}{\rho d^4(1-\nu^2)}} \quad ; \text{where, } n = 2 \quad \text{for 11 mode} \tag{3}$$

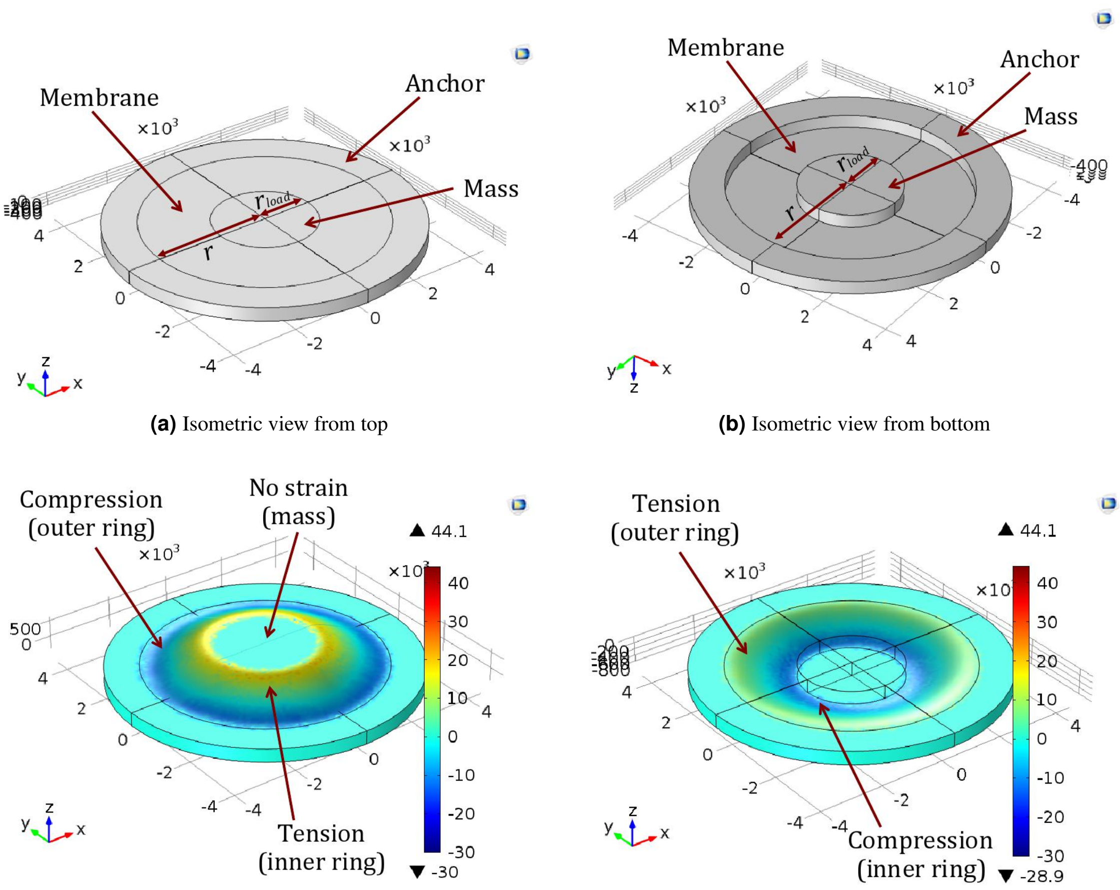

(a) Isometric view from top **(b)** Isometric view from bottom

(c) Volumetric strain for 1st half oscillatory cycle **(d)** Volumetric strain for 2nd half oscillatory cycle

Figure 1. COMSOL design model and FEA simulation of the volumetric strain during oscillation for a MEMS disk membrane resonator. The membrane is the silicon device layer while the suspended mass is constructed from the unetched silicon substrate.

Plate stiffness factor D is given by equation 4.[5]

$$D = \frac{Eh^3}{12(1-\nu^2)} \tag{4}$$

For a massless circular disk membrane with concentrated centred mass, the natural frequency can be represented by equation 5.[5] Substituting in equation 4, leads to equation 6.[4]

$$\omega_n = 7.084\sqrt{\frac{D}{mr^2}} \tag{5}$$

$$\omega_n = 4.09\sqrt{\frac{Eh^3}{md^2(1-\nu^2)}} \tag{6}$$

Therefore, assuming the concentrated mass is the membrane mass, a circular disk membrane with significant plate mass can be derived as shown in equation 8.

$$\omega_n = 4.09\sqrt{\frac{Eh^3}{(0.152\pi r^2 h\rho)d^2(1-\nu^2)}} \tag{7}$$

$$\omega_n = 2.045\sqrt{\frac{Eh^3}{(0.152m_{disk})r^2(1-\nu^2)}} \tag{8}$$

Furthermore, by combining equations 6 and 8, a circular disk membrane with both significant plate mass and concentrated centred mass can be represented by equation 9.

$$\omega_n = 2.045\sqrt{\frac{Eh^3}{(m_{load}+0.152m_{disk})r^2(1-\nu^2)}} \tag{9}$$

However, for practical devices with physical dimensions, a concentrated mass approximation for the proof mass or added load is ineffective. Therefore, an effective length or radius of the proof mass needs to be calculated similar to that of a cantilever beam with a proof mass. Assuming the radius contribution of the proof mass is around half of the mass, the effective radius r_{eff} of a circular plate membrane with added circular centre mass of radius r_{load} and thickness h_{load} can be derived as shown in equations 10 to 12.

$$\pi r_{m/2}^2 h_{load}\rho = \frac{1}{2}\pi r_{load}^2 h_{load}\rho \tag{10}$$

$$r_{m/2} = \frac{1}{\sqrt{2}} r_{load} \tag{11}$$

$$r_{eff} = r - \frac{r_{load}}{\sqrt{2}} \tag{12}$$

Henceforth, assuming effective radius r_{eff}, the adjusted natural frequency of a circular membrane with significant plate mass and a circular centred mass is shown in equation 13.

$$\omega_n = 2.045\sqrt{\frac{Eh^3}{\pi\rho(r_{load}^2 h_{load}+0.152r^2h)(r-\frac{r_{load}}{\sqrt{2}})^2(1-\nu^2)}} \tag{13}$$

Simulation

Figure 2 shows the numerically simulated response for the first order parametric resonance for a system with parameters: $\omega_n = 5.0513\text{E}3$ rads^{-1}, $\xi = 1.7744\text{E}6$, ζ_1 =0.05, $\zeta_2 = 7.3934\text{E}3$ and μ = -0.01 (representative of a MEMS device). The Runga-Kutta numerical solver ode4 in MATLAB was employed with a fixed step size of 0.5 μs. The simulated result for the second order and third order parametric resonance is included in the supplementary document.

Figures 3 and 4 show the simulated response of 2nd and 3rd order parametric resonance, with frequency frequency always at the natural frequency. Note that the response frequency for nth order is precisely 0.5n times the excitation frequency.

Apparatus

Figure 5 shows the fabricated MEMS chip (12 mm by 12 mm) fixed to a leadless chip carrier (LCC) using an epoxy adhesive. The LCC was back etched using laser micro-machining in order to accommodate the proof mass of the resonator to freely travel without physical constraints.

The MEMS chip, assembled on the the ceramic chip carrier and electrically wire-bonded to a measurement circuit, is mechanically fixed onto a vibration platform. An accelerometer was also mechanically mounted alongside the MEMS harvester device to characterise the vibratory excitation and further details on the experimental setup and method are included in the supplementary document.

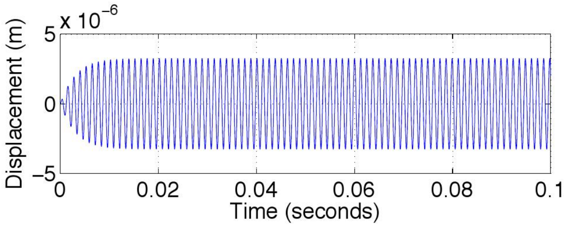

(a) Direct resonant (1st mode) build up $\omega = \omega_n$

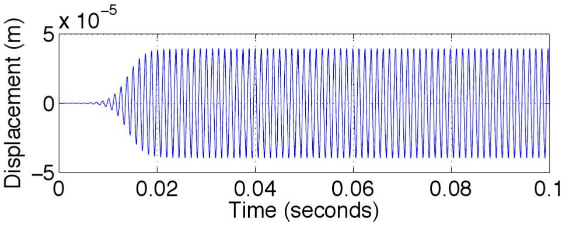

(b) Parametric resonant (1st order) build up $\omega = 2\omega_n$

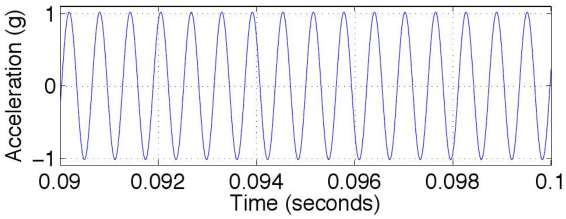

(c) Parametric input frequency $\omega = 2\omega_n$

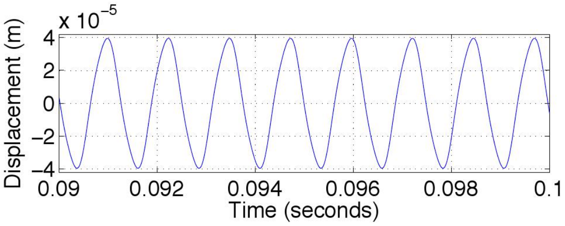

(d) Parametric resonant response frequency at ω_n

Figure 2. Numerically simulated direct resonant response and the first order parametric resonant response.

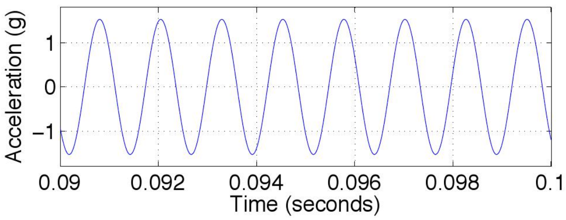

(a) Input frequency $\omega = \omega_n$

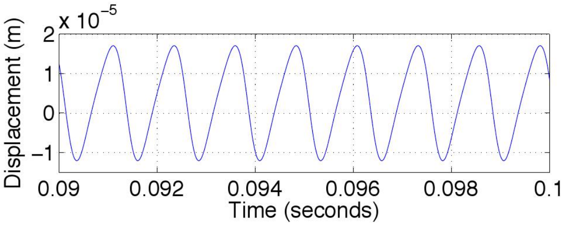

(b) Response frequency at ω_n

Figure 3. Numerically simulated second order parametric resonance.

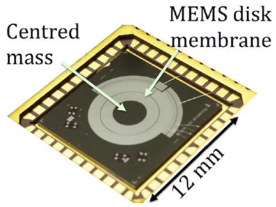

(a) Top side

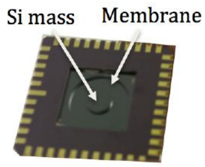

(b) Back side

Figure 5. MEMS chip, attached to a laser holed leadless chip carrier to allow free travel of disk resonator mass.

Figure 6 shows the experimental setup where the MEMS chip is assembled within a ceramic chip carrier and electrically wire-bonded. The carrier is than mechanically assembled on a vibration platform.

The overview of the experimental apparatus is presented in figure 7.

Experimental Results

Higher orders

The electrically undamped response of the resonator was recorded by measuring the open circuit output on the oscilloscope. Additionally, the power response was characterised by measuring the voltage response across a matched resistive load of 70 kΩ. This optimal load determination is valid for response in the vicinity of the natural frequency of 980 Hz.

The details of the excitation frequency range of all 28 orders are delineated in table 1.

The principal (first order) parametric resonance was the easiest of all the orders to be activated with a drive frequency

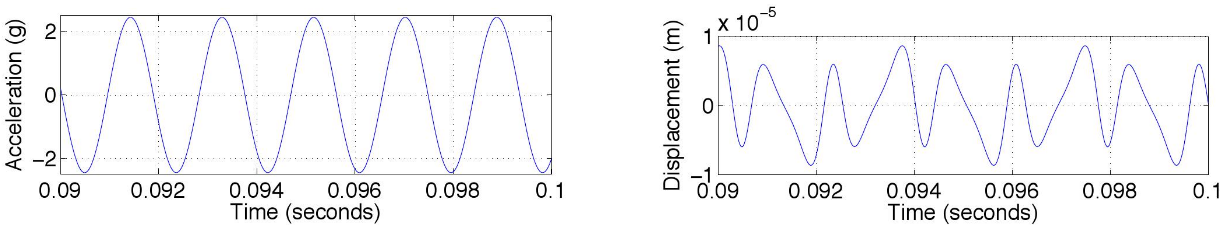

(a) Input frequency $\omega = 2\omega_n/3$ **(b)** Response frequency at ω_n

Figure 4. Numerically simulated third order parametric resonance.

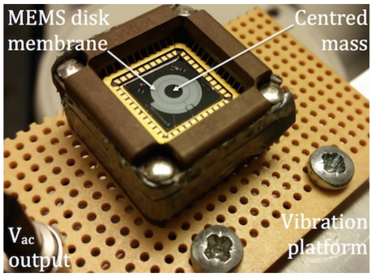

Figure 6. Photograph of the MEMS chip, housed inside a chip carrier and socket, which is mounted on a vibration platform.

of twice the natural frequency at around 2 kHz. Once operating inside the instability region, sweeping the drive frequency downwards, the first order was maintained down to 1.6 kHz. However, activating the first order at 1.6 to 1.8 kHz from outside the instability region required significantly higher acceleration than what was required to maintain operation within the instability region. Additionally fast modification of the drive amplitude or frequency can push the system out of the instability region, even if the excitation conditions are still theoretically favourable. This illustrates the strict boundary conditions required to activate parametric resonance.

The second order parametric resonance and the first mode direct resonance have the same excitation and response frequencies. The second order is differentiated by sudden amplitude jumps between frequency bands (instability region boundaries) and critical amplitude values (initiation threshold amplitudes). This is because upon exiting the instability region, the system experiences an abrupt decay in amplitude.

The modified instability charts of the MEMS resonator in the results section were measured by first activating the parametric resonance and then gradually reducing the excitation to the minimum acceleration level required to sustain the respective parametric resonant condition. This corresponds to the theoretical minimum required to access the instability region. Therefore in practice, a higher acceleration level is necessary to push the oscillatory system into parametric resonance.

Vibration energy harvesting

The power and bandwidth values included here shows an example of what the MEMS membrane device can produce at a particular acceleration level. At 1.0 g of vibrational excitation, a peak power output of 1.58 μW and half power bandwidth of 55 Hz was recorded for the 1st mode direct resonance, 3.46 μW for the 1st order parametric resonance, 3.4 μW for the 2nd order,

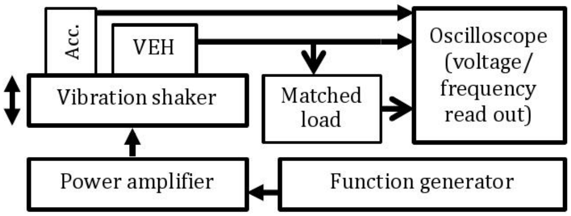

Figure 7. Outline of the experimental apparatus setup. Acc. is an off-the-shelf MEMS accelerometer. The output from the vibration energy harvester (VEH) can either be directly monitored on an oscilloscope for its open circuit (electrically undamped) output or connected in parallel to a matched resistive load for its matched power output.

Table 1. Measured frequency range for all 28 orders of parametric resonance fall within the vicinity of the theoretically predicted frequency points. Natural frequency is 980 Hz and all values are for excitation frequency.

Order number	Theory (Hz)	Measured frequency (Hz) range	width
1	1960	1600 to 2100	500
2	980	730 to 1000	270
3	653	610 to 720	110
4	490	430 to 500	70
5	392	360 to 409	49
6	327	302 to 330	28
7	280	264 to 286	22
8	245	240 to 260	20
9	218	215 to 234	19
10	196	192 to 210	18
11	178	168 to 180	12
12	163	157 to 165	8
13	151	146 to 152	6
14	140	136 to 141	5
15	131	127.0 to 131.4	4.4
16	123	120.7 to 123.6	2.9
17	115	113.0 to 115.5	2.5
18	109	107.0 to 109.2	2.2
19	103	101.9 to 103.7	1.8
20	98	97.0 to 98.6	1.6
21	93	92.1 to 93.5	1.4
22	89	88.2 to 89.4	1.2
23	85	84.3 to 85.3	1.0
24	82	81.2 to 82.4	1.2
25	78	77.4 to 78.1	0.7
26	75	74.6 to 75.2	0.6
27	73	72.8 to 73.1	0.3
28	70	69.92 to 70.04	0.12

2.5 μW for the 3rd order, 0.8 μW for the 4th order, 0.2 μW for the 5th order were measured across a matched load of 70 kΩ.

Parametric excitation of the first three orders provided higher peak output power than the corresponding peak output power for the direct resonant response, while the first two orders had wider half power bandwidth (314 Hz and 135 Hz respectively) than their direct counterpart (55 Hz). Although observable, both the amplitude and frequency bandwidth of the 4th and 5th order parametric resonance were significantly smaller in comparison to the 1st mode direct resonance. Nonetheless, the presence of the higher orders help open up multi-frequency responsive bands, which would otherwise be non-existent in the absence of parametric resonance. Therefore, this contributes to the significantly larger accumulative half power bandwidth (478 Hz) of the resonator for the purpose of VEH.

References

1. Hong, E., Trolier-McKinstry, S., Smith, R., Krishnaswamy, S. V. & Freidhoff, C. B. Vibration of micromachined circular piezoelectric diaphragms. *IEEE Trans. Ultrason., Ferroelect., Freq. Control.* **53**, 697–706 (2006).
2. Novak, S. & Frehlich, R. G. Transition to chaos in the duffing oscillator. *Phys Rev A* **26**, 3660–3 (1982).
3. Turner, K. et al. Five parametric resoannces in a microelectromechanical system. *Nature* **396**, 149–152 (1988).
4. Piersol, A. G. & Paez, T. L. *Shock and Vibrations Handbook* (McGraw Hill, 2009), 6 edn.
5. Pilkey, W. D. *Formulas for stress, strain, and structural matrices* (Wiley, 2005).

Acknowledgement

This work was supported by EPSRC (Grant EP/L010917/1).

Author contributions statement

YJ and AAS conceived the research; YJ designed the MEMS devices and the experiment; YJ and SD performed the experiments; YJ and AAS developed device models. All authors contributed to analysing the results as well as writing and reviewing of the manuscript.

Competing financial interests

YJ and AAS are co-founders of 8power Ltd.
